# Identification and characterization of *Burkholderia multivorans* CCA53

**DOI:** 10.1186/s13104-017-2565-1

**Published:** 2017-07-06

**Authors:** Hironaga Akita, Zen-ichiro Kimura, Mohd Zulkhairi Mohd Yusoff, Nobutaka Nakashima, Tamotsu Hoshino

**Affiliations:** 1 Research Institute for Sustainable Chemistry, National Institute of Advanced Industrial Science and Technology (AIST), 3-11-32 Kagamiyama, Higashi-Hiroshima, Hiroshima 739-0046 Japan; 2 Department of Civil and Environmental Engineering, National Institute of Technology, Kure College, 2-2-11 Aga-minami, Kure, Hiroshima 737-8506 Japan; 3 Department of Bioprocess Technology, Faculty of Biotechnology and Biomolecular Sciences, Universiti Putra Malaysia, 43400 Serdang, Selangor Malaysia; 4 Bioproduction Research Institute, National Institute of Advanced Industrial Science and Technology (AIST), 2-17-2-1 Tsukisamu-Higashi, Toyohira-ku, Sapporo, Hokkaido 062-8517 Japan; 5 Department of Biological Information, Graduate School of Bioscience and Biotechnology, Tokyo Institute of Technology, 2-12-1-M6-5 Ookayama, Meguro-ku, Tokyo, 152-8550 Japan

**Keywords:** *Burkholderia multivorans*, MLST analysis, Lignin-degrading bacterium, Second-generation biofuel

## Abstract

**Objective:**

A lignin-degrading bacterium, *Burkholderia* sp. CCA53, was previously isolated from leaf soil. The purpose of this study was to determine phenotypic and biochemical features of *Burkholderia* sp. CCA53.

**Results:**

Multilocus sequence typing (MLST) analysis based on fragments of the *atpD*, *gltD*, *gyrB*, *lepA*, *recA* and *trpB* gene sequences was performed to identify *Burkholderia* sp. CCA53. The MLST analysis revealed that *Burkholderia* sp. CCA53 was tightly clustered with *B. multivorans* ATCC BAA-247^T^. The quinone and cellular fatty acid profiles, carbon source utilization, growth temperature and pH were consistent with the characteristics of *B. multivorans* species. *Burkholderia* sp. CCA53 was therefore identified as *B. multivorans* CCA53.

## Introduction

The genus *Burkholderia* was firstly proposed by Yabuuchi et al. [1], and was classified as Gram-negative and non-spore forming β-proteobacteria. To date, more than 80 *Burkholderia* species have been reported, and two major clusters and several subgroups have been proposed based on phylogenetic analyses of the 16S rRNA, *acd*, *gyrB*, *recA* and *rpoB* gene sequences, as well as their genome sequences [2]. Group A contains plant-associated and saprophytic species [2]. For example, nitrogen fixation in legumes is facilitated by *B. mimosarum*, *B. nodosa*, *B. sabiae*, *B. tuberum* and *B. phymatum* [3]. Also, growth rates of a few plants are promoted by *B. phytofirmans* and *B. unamae* [3]. On the other hand, group B contains opportunistic pathogens that infect animals, humans and plants [2]. *B. cenocepacia*, *B. latens* and *B. multivorans* infect to cystic fibrosis patients, which leads to pneumonic illness [4, 5]. *B. cenocepacia*, *B. multivorans* and *B. vietnamiensis* show infectivity to alfalfa and lettuce [6].

Several *Burkholderia* species are now being utilized in industrial applications as biocatalysts [7, 8], for biodegradation [9] and as plant growth-promoting rhizobacteria [3]. For example, *B. fungorum* DBT1 is capable of assimilating polycyclic aromatic hydrocarbons, which is useful for bioremediation of contaminated soils [10]. *B. cepacia* GS3C exhibits highly efficient degradation during bioremediation of oil-contaminated soil [11, 12], and *B. cepacia* PCL3 is useful for treating carbofuran-contaminated water [13]. In addition, several antibiotics, including cepaciamide A [14], glidobactin A [15], pyrrolnitrin [16] and xylocandins [17] are produced by *Burkholderia* species. Several *Burkholderia* species showed lignin degradation capabilities [18], which are favorable to produce second-generation biofuels from lignocellulosic biomass. Thus, *Burkholderia* species are versatile bacteria with potential applicability in the biochemical and pharmaceutical industries. We previously isolated *Burkholderia* sp. CCA53 from leaf soil [19] and determined the draft genome sequence of the strain [20]. In this study, we report the phenotypic and biochemical characterization of *Burkholderia* sp. CCA53.

## Main text

### Methods

MLST analysis was performed according to the method of Urwin and Maiden [21]. A phylogenetic tree of concatenated sequences (9348 bp), including fragments of six housekeeping genes [*atpD* (1380 bp), *gltD* (1467 bp), *gyrB* (2469 bp), *lepA* (1794 bp), *recA* (1044 bp), *trpB* (1194 bp)] from *Burkholderia* sp. CCA53, was reconstructed based on the neighbor-joining method [22]. The calculation of distances, multiple alignment and construction of neighbor-joining phylogenetic trees were performed using CLUSTAL W version 1.83 [23]. All gene sequences are available in the GenBank/EMBL/DDBJ databases under the accession numbers BDDJ01000001 to BDDJ01000004.

*Burkholderia* sp. CCA53 (strain number: HUT-8135) was cultured in Nutrient Broth (Kyokuto, Tokyo, Japan). The OD_600_ was monitored by measuring the difference between the cell and cell-free culture turbidity values using an Eppendorf BioSpectrometer (Eppendorf, Hamburg, Germany). Carbon source utilization was determined using API 20E (bioMérieux, Marcy-l’Etoile, France) and API 50CHE (bioMérieux) according to the manufacturer’s instructions. The effects of temperature (10–60 °C) and pH (3.0–10.0) on the growth were studied.

The lipid was extracted from lyophilized cells according to the method of Bligh and Dyer [24], and then loaded onto a Sep-Pak Plus Silica cartridge (Waters, Milford, MA, USA). After washing the cartridge, the quinone was eluted. Quinone quantification was performed using an ACQUITY UPLC system (Waters) with an Eclipse Plus C18 column (Agilent technologies, Santa Clara, CA, USA). The chromatographic conditions were as follows: mobile phase, methanol/isopropanol (3:1 v/v); flow rate, 0.5 mL min^−1^; the column oven temperature, 35 °C. The identification of quinone forms was carried out as previously described [25].

The cellular fatty acid compositions were determined using the Sherlock Microbial Identification System Version 6.0 (MIDI, Newark, DE, USA) and TSBA6 database (MIDI).

### Results

**Fig. 1:**
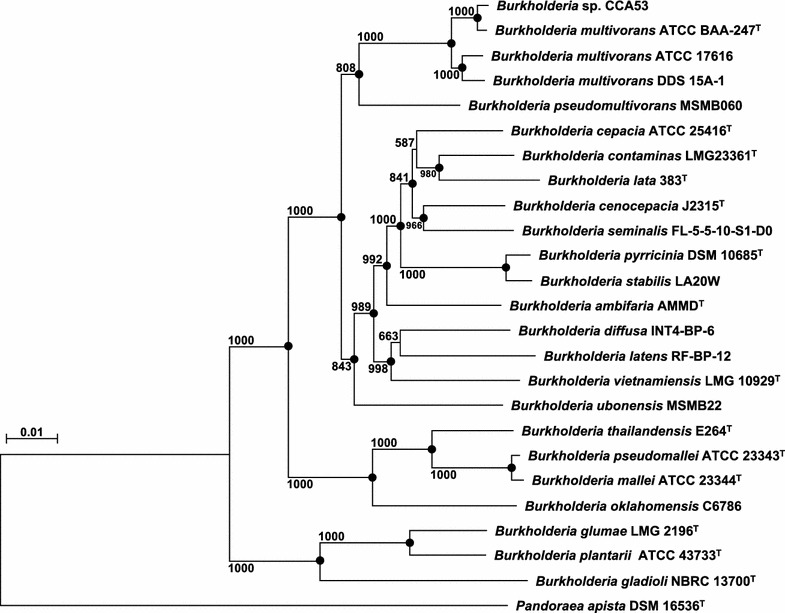
Phylogenetic tree reconstructed from analysis of the sequences of six housekeeping genes (*atpD*, *gltD*, *gyrB*, *lepA*, *recA* and *trpB*) and showing the relationship of CCA53 with related species. *Pandoraea apista* DSM 16536^T^ was used as an outgroup. The tree was reconstructed using the neighbor-joining method with Kimura’s two-parameter model [28]. *Closed circles* show the nodes supported by 80% bootstrap probabilities with 1000 replicates. The *bar* indicates the 1% nucleotide substitution rate

Using MLST analysis with housekeeping genes, several *Burkholderia* species were identified. For example, the existence of *Burkholderia cepacia* complex species in moso bamboo plantations [26] and water bodies [6] were determined by MLST analysis based on fragments of the *atpD*, *gltBD*, *gyrB*, *lepA*, *recA*, *phaC* and *trpB* gene sequences. Moreover, *Burkholderia* phylogeny was revealed by rMLST, which was constructed based on the ribosomal protein-encoding genes of *Burkholderia* species [27]. To identify the phylogeny of *Burkholderia* sp. CCA53, we also performed an MLST analysis based on fragments of the *atpD*, *gltD*, *gyrB*, *lepA*, *recA* and *trpB* gene sequences (Fig. 1). The form of the resultant phylogenetic tree was similar to those of MLST [6, 26]. The MLST analysis showed that *Burkholderia* sp. CCA53 shared a high degree of similarity with *B. pseudomultivorans* MSMB060 (95.7%) and *B.ubonensis* MSMB22 (94.0%). Moreover, *Burkholderia* sp. CCA53 was closely related to *B. multivorans* ATCC BAA-247^T^ (99.6%), ATCC 17616 (98.7%) and DDS 15A-1 (98.7%). Thus, *Burkholderia* sp. CCA53 was identified as *B. multivorans* CCA53.

When *B. multivorans* CCA53 was cultured aerobically in Nutrient Broth, ubiquinone-8 was detected as the major respiratory quinone (98.7%), and a small amount of ubiquinone-9 was also detected (1.3%). This suggests that ubiquinone-8 exclusively functions in the quinone system of *B. multivorans* CCA53, which is consistent with the quinone profiles of *B. kururiensis* [29], *B. megalochromosomata* [30] and *B. uboniae* [31].

The following fatty acids were present in *B. multivorans* CCA53: C_12:0_ (0.1%), C_13:1_ (0.7%), C_14:0_ (4.2%), C_14:0_ 2-OH (0.2%), C_16:0_ (24.0%), C_16:0_ 2-OH (1.8%), C_16:0_ 3-OH (6.0%), C_16:1_ 2-OH (1.3%), C_17:0_ (0.4%), anteiso-C_17:0_ ω9c (0.1%), cyclo-C_17:0_ (8.4%), C_18:0_ (1.5%), C_18:1_ ω5c (0.1%), 11-methyl-C_18:1_ ω7c (0.1%), cyclo-C_19:0_ ω8c (9.0%), iso-C_19:0_ (0.2%), summed feature 2 (comprising C_14:0_ 3-OH, and/or iso-C_16:1_ I, and/or C_12:0_ unidentified aldehyde or an unidentified fatty acid with equivalent chain length of 10.928) (5.8%), summed feature 3 (comprising C_16:1_ ω6c and/or C_16:1_ ω7c) (11.6%) and summed feature 8 (comprising C_18:1_ ω6c and/or C_18:1_ ω7c) (24.6%). The unsaturated fatty acids C_16:1_ ω6c, C_16:1_ ω7c, C_18:1_ ω6c and C_18:1_ ω7c were major components of *B. multivorans* CCA53. This fatty acid profile conformed to the profiles of *B. multivorans* ATCC 17616 [32] and *B. multivorans* CGD2 [32].

To determine its carbon source utilization, *B. multivorans* CCA53 was cultured with each carbon sources. This revealed that *B. multivorans* CCA53 utilized the following compounds as carbon sources for growth: amygdalin, d-lactose, d-maltose, d-cellobiose, d-arabinose, l-arabinose, d-fucose, d-fructose, d-galactose, d-glucose, d-mannose, d-ribose, d-xylose, d-adonitol, d-arabitol, l-arabitol, dulcitol, inositol, d-mannitol, d-sorbitol, l-arginine, l-lysine, l-ornithine, l-tryptophane, citrate, pyruvate and urea. Among those, assimilation of d-galactose, d-glucose, d-mannose, d-xylose, d-adonitol, inositol and d-sorbitol is common to *Burkholderia* species [1]. On the other hand, no growth occurred on gelatin, glycogen, starch, inulin, d-melezitose, d-raffinose, arbutin, esculin ferric citrate, gentiobiose, d-melibiose, 2-nitrophenyl β-d-galactopyranoside, salicin, d-sucrose, d-trehalose, d-turanose, *N*-acetyl-glucosamine, l-fucose, d-lyxose, methyl-α-d-glucopyranoside, methyl-α-d-mannopyranoside, methyl-β-d-xylopyranoside, l-rhamnose, l-sorbose, d-tagatose, l-xylose, gluconate, 2-keto gluconate, 5-keto gluconate, d-mannito, xylitol, erythritol, glycerol or thiosulfate.

**Fig. 2:**
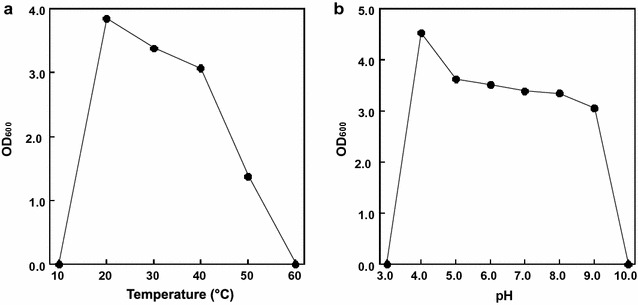
Effects of temperature and pH on growth of *B. multivorans* CCA53. Cells were cultured in Nutrient Broth. *Error bars* indicate SE (*n* = 3)

When *B. multivorans* CCA53 was cultured in Nutrient Broth at various temperatures (10–60 °C), the maximum growth rate was achieved at 20 °C (Fig. 2a). The strain was capable of growing at temperatures between 20 and 50 °C, but no growth was seen at 60 °C (Fig. 2a). At 30 °C, the maximum growth rate of *B. multivorans* CCA53 was at pH 4.0 (Fig. 2b). Moreover, the strain grew effectively at pHs between 4.0 and 9.0, but growth rates were sharply lower at pHs below 3.0 or above 10.0 (Fig. 2b). These characteristics were nearly the same as those of *B. multivorans* NKI379, which was also isolated from soil samples in the Er-Ren River Basin, Taiwan [33].

### Discussion

From the viewpoints of economics, ecology and environmental protection, it would be advantageous to produce biofuels from lignocellulosic biomass, which are known as second-generation biofuels [34]. When second-generation biofuels are produced from lignocellulosic biomass, consecutive pretreatment, enzymatic hydrolysis and microbial fermentation steps are required. During the pretreatment step, lignocellulosic biomass is decomposed through heating, which releases cellulose, hemicellulose and lignin. At the enzymatic hydrolysis step, cellulose and hemicellulose are converted into saccharified solution, which includes fermentable sugars, aldehyde inhibitors and lignin. In the fermentation step, the fermentable sugars are used as carbon sources by engineered *Escherichia coli*, *Saccharomyces cerevisiae* or other microorganisms [35, 36]. Although aldehyde inhibitors inhibit microbial growth and interfere with subsequent fermentation, these compounds can be chemically or enzymatically detoxified [34, 37]. However lignin is not effectively utilized by the aforementioned microorganisms, causing the yield to be low [35, 36]. Microbial degradation of lignin has been primarily studied in brown- and white-rot fungi. Using the Fenton reaction, brown-rot fungi produce free hydroxyl radicals from hydrogen peroxide, after which the free hydroxyl radicals are used in the lignin degradation [38]. Moreover, white-rot fungi are capable of producing several extracellular ligninolytic enzymes, including laccase, lignin peroxidase, manganese peroxidase and versatile peroxidase, which are also useful for lignin degradation [39]. On the other hand, these fungi show slower growth rates and require for long incubation times, which elevates the production costs and draws lower productivities. A few bacterial species belonging to the genera *Arthrobacter*, *Burkholderia, Comamonas*, *Pseudomonas*, *Sphingobium*, *Streptomyces* and *Rhodococcus* show faster growth rates and lignin degradation capabilities, but their capabilities are lower than those of fungi [18]. We therefore screened for lignin-degrading bacteria with rapid growth rates and high capabilities for lignin degradation, and a candidate bacterium was isolated from leaf soil [19]. Based on its 16S rRNA gene sequence homology, the bacterium was identified as *Burkholderia* sp. CCA53 [19]. This strain was capable of utilizing lignin as a sole carbon source, and it was anticipated that *Burkholderia* sp. CCA53 would have industrial potential for second-generation biofuel production [19]. In the present study, therefore, we characterized the phenotypic and biochemical features of *Burkholderia* sp. CCA53. Several *Burkholderia* species, including *B. cepacia* KK01 [40] and *Burkholderia* sp. LIG30 [41] also have a capacity to degrade lignin. In *Burkholderia* sp. LIG30, the mechanism of its lignin degradation is suggested by its expression of two genes predicted to encode multi-copper oxidase and 22 genes encoding putative catalases or peroxidases [41]. Within the draft genome sequence of *B. multivorans* CCA53, one gene predicted to encode multi-copper oxidase and 21 genes encoding putative catalases or peroxidases were also confirmed [20]. This suggests the mechanism for lignin degradation used by *B. multivorans* CCA53 may be similar to that used by *Burkholderia* sp. LIG30.

When saccharified solutions are prepared from sugarcane, cassava and their wastes, d-glucose and l-xylose are the main saccharides [42, 43], though small amounts of d-lactose and d-maltose are also present [42, 43]. Several *Burkholderia* species cannot assimilate d-lactose or d-maltose [29], but *B. multivorans* CCA53 was able to use all of these disaccharides as carbon sources, which means that *B. multivorans* CCA53 could be a useful strain for production of second-generation biofuels [35, 36]. Moreover, we think that *B. multivorans* CCA53 may have other advantages for industrial application beyond utilization of lignin. The first is that *B. multivorans* CCA53 showed efficient growth at acidic pH (Fig. 2b). Several lignocellulosic biomass-degrading enzymes showed maximum activities at acidic pH [44–46], which means that the saccharified solution pH is also acidic. By contrast, the growth of industrial bacteria such as *E. coli* is inefficient at acidic pH. Consequently, pH control is required at the fermentation step with engineered *E. coli*, whereas *B. multivorans* CCA53 would not require pH control. Second, the optimal growth pH for *B. multivorans* CCA53 would be expected to prevent contamination by microorganisms in larger scale fermentations. Third, *B. multivorans* CCA53 showed strong growth at 20–40 °C (Fig. 2a), which is similar to the mesophilic conditions required for *E. coli* and *S. cerevisiae*. This means that the existing systems for biofuel fermentation will be applicable for use with *B. multivorans* CCA53.

### Limitations

In this paper, we reported the phylogenetic, phenotypic and biochemical characterization of *Burkholderia* sp. CCA53. To identify the phylogeny of *Burkholderia* sp. CCA53, we performed MLST analysis. In addition, results of phenotypic and biochemical analyses were consistent with the characteristics of *B. multivorans* species. *Burkholderia* sp. CCA53 was therefore identified as *B. multivorans* CCA53. These results may give little interest for microbiologists.

## Abbreviation

MLST: multilocus sequence typing
